# Perinatal tuberculosis—An approach to an under-recognized diagnosis

**DOI:** 10.3389/fpubh.2023.1239734

**Published:** 2023-11-07

**Authors:** H. Simon Schaaf, Adrie Bekker, Helena Rabie

**Affiliations:** ^1^Desmond Tutu TB Centre, Department of Paediatrics and Child Health, Faculty of Medicine and Health Sciences, Stellenbosch University, Cape Town, South Africa; ^2^Department of Paediatrics and Child Health, Faculty of Medicine and Health Sciences, Stellenbosch University, Cape Town, South Africa

**Keywords:** tuberculosis, perinatal, congenital, postnatal, prevention, diagnosis, treatment

## Abstract

Tuberculosis (TB) in young infants (<3 months of age), often referred to as perinatal TB, is underdiagnosed, leading to severe morbidity and high mortality. Perinatal TB includes both congenital and postnatal transmission of *Mycobacterium tuberculosis*. We aimed to increase an awareness of TB in neonates and young infants and to provide guidance on the assessment and management when in contact with mothers with TB during or soon after pregnancy. Approximately 217,000 pregnant women develop TB annually; if they are not diagnosed and treated during pregnancy, their infants are at high risk of adverse birth outcomes and TB disease. Although safe and effective antituberculosis treatment regimens are available during pregnancy, the diagnosis of TB is challenging. Infants born to mothers newly diagnosed with TB, not receiving any effective treatment or with cultures not yet negative, should be assessed for TB disease or *M. tuberculosis* infection. TB preventive therapy should be instituted if the infant is clinically well but exposed to TB, while prompt initiation of TB treatment is essential if TB disease is presumed. HIV status of mother and infant should be considered as this will affect the management. Further research is needed for the diagnosis and prevention of TB during pregnancy, an early diagnosis of TB in infants, and antituberculosis drug pharmacokinetics in young infants.

## Introduction

Tuberculosis (TB) remains the infectious disease with the highest mortality globally. In 2021, an estimated 10.6 million new TB cases occurred, including 1.17 million children. Of the 1.59 million deaths in 2021, 14% were in children aged 0–14 years (1). Mathematical models for 2015 estimated that of the 239,000 (95% uncertainty interval [UI]: 194,000–298,000) TB-related deaths in children aged 0–14 years, 191,000 (80%; 95% UI 132,000–257,000) occurred in children younger than 5 years of age. The per-person TB mortality in children is highest in sub-Saharan African countries with high annual TB incidences and almost all (228,000; 96%) deaths occurring in children not on treatment (2).

Immune immaturity related to young age is associated with a high risk of developing TB disease following infection. Infants (< 12 months) experience the highest risk of developing TB (50%) after infection in the absence of preventive measures; among these, up to 30% develop progressive pulmonary or disseminated (miliary) TB (3). Although vertical transmission prevention programmes are successful in preventing perinatal and breastfeeding-related HIV infection, infants born to mothers living with HIV remain at an increased risk of exposure to TB because of the high TB prevalence in people living with HIV (4, 5). Maternal TB also increases the risk of HIV transmission (5).

TB in infants of <3 months of age can either be congenital or postnatal in origin. It is challenging to clinically distinguish between congenital TB, transmitted *in utero* by haematogenous spread through the umbilical vein or ingestion/aspiration of *Mycobacterium tuberculosis*-infected amniotic fluid during birth, and postnatal transmission that occurs by the inhalation of *M. tuberculosis* bacilli spread by the airborne route from a mother or other close source patient with infectious pulmonary TB early after birth; therefore, the term perinatal TB is preferred (i.e., infants likely infected congenitally or within the 1^st^ days of life postnatally and presenting within the first 3 months of life) (6). The focus of this minireview was to increase awareness of TB in neonates (<28 days) and young infants (<3 months), describe the clinical presentation, and provide guidance on the investigation and the management of young infants in contact with mothers with TB during or after pregnancy.

## Epidemiology

Due to immunological changes, women are at higher risk of developing TB disease after *M. tuberculosis* infection during pregnancy and in the immediate postpartum period (7). HIV infection further increases this risk (6–8). A review in 2012 estimated the TB prevalence among pregnant women to range from 0.06–0.25% in low-burden countries to 0.07–0.5% in high-burden countries (9). The documented rates were 0.07–0.5% in HIV-negative women and 0.7–11% in HIV-positive women (9). Epidemiological modeling estimated that in 2011, there were 216,500 (95% uncertainty range 192,000–247,000) pregnant women with TB globally (10).

TB in pregnancy is associated with poor pregnancy outcomes, and undiagnosed or untreated TB during pregnancy and postpartum holds a high risk for perinatal *M. tuberculosis* infection and disease in infants. Compared with pregnant women without TB, pregnant women with TB disease had an increased risk of preterm birth (OR 1.7, 95% CI 1.2–2.4), giving birth to low birth weight infants (OR 1.7, 95% CI 1.2–2.4), birth asphyxia (OR 4.6, 95% CI 2.4–8.6), and perinatal death (OR 4.2, 95% CI 1.5–11.8) (11). The multitude of case reports, several case series, the likelihood that many cases are undiagnosed and not reported, and data from high HIV-burden settings suggest that congenital TB is not as rare as most reports suggest (6, 7). However, postnatal transmission of *M. tuberculosis* remains more common than congenital TB and in the first 3 months of life often presents with severe disease because of the immature immune system. Over a 12.3-year period in a high TB-incidence setting, 106 of 2017 (5%) children under 13 years of age with culture-confirmed TB were infants under 3 months of age (12).

Possible risk factors for congenital TB are as follows: (1) Infants born to mothers who have primary TB, which often has a haematogenous (bacillaemic) phase (see Clinical presentation) (6), (2) mothers co-infected with TB and HIV, and (3) mothers who had undergone *in vitro* fertilization (IVF) with TB as the cause of infertility (13).

## Prevention of TB in mothers and their infants

Prevention of TB disease in pregnant women and prevention of TB in infants of pregnant women with TB are paramount. This has four components: (1) primary prevention of TB among pregnant women, especially by providing TB preventive therapy (TPT) after infection or high-risk TB exposure (14, 15); (2) preventing unintended pregnancies in women receiving TB therapy; (3) early identification and treatment of TB with treatment support in women during pregnancy and postpartum; and (4) providing appropriate post-TB-exposure TPT to infants when indicated (16). The risk of untreated TB disease to the pregnant woman and her fetus is much greater than the risks of antituberculosis treatment. Appropriate and safe regimens for both drug-susceptible and drug-resistant TB treatment in pregnancy are available (15).

TB of the genitourinary tract is a common cause of infertility; several cases of congenital TB were reported in infants of mothers who had undergone IVF; therefore, TB should be excluded and treated before IVF is performed to prevent congenital TB in their infants (13).

### TB preventive therapy in exposed/infected neonates and young infants

**Figure 1:**
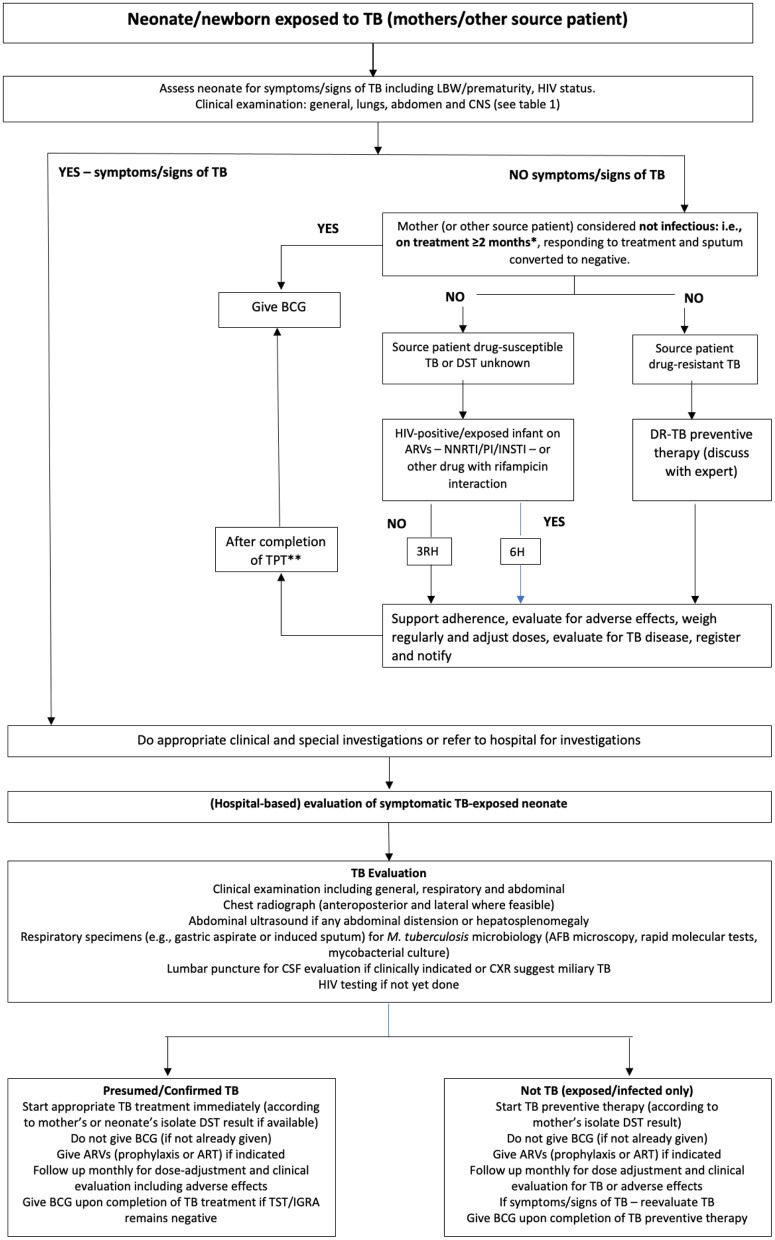
An algorithm for the assessment and management of newborn infants/neonates in contact with mothers (or other source cases) with tuberculosis during pregnancy or directly after birth. (Adapted from Van der Westhuizen et al. (17) and National Department of Health, South Africa (18) with permission). *Some experts recommend mothers to only be on treatment for 2 weeks during pregnancy, but this duration is insufficient to prevent congenital exposure and postnatal exposure if still microbiologically positive for *Mycobacterium tuberculosis* on sputum. **If tuberculin skin test (or interferon-gamma release assay) is negative or not available. AFB, acid-fast bacilli; ARV, antiretroviral; ART, antiretroviral treatment; BCG, Calmette–Guérin; CNS, central nervous system; CXR, chest x-ray; DR-TB, drug-resistant tuberculosis; DST, drug susceptibility testing; INSTI, integrase strand transfer inhibitor; LBW, low birth weight; *M. tuberculosis*, Mycobacterium tuberculosis; NNRTI, non-nucleoside reverse transcriptase inhibitor; PI, protease inhibitor; TPT, tuberculosis preventive therapy; 3HR, 3 months of isoniazid plus rifampicin; 6H, 6 months of isoniazid.

When mothers are diagnosed and/or treated for TB in pregnancy, their neonates should be screened for TB at birth or within the 1st days of life (Figure 1) (17–19). However, many young infants with TB are the index (first diagnosed) patients, and after their diagnosis, a source case should be sought. In most cases, this is the mother who may have been asymptomatic in pregnancy, previously undiagnosed, or her symptoms were not identified as related to possible TB (20, 21).

In neonates screened for TB with known exposure or infection, TPT should be provided once TB disease has been excluded (15, 22).

The choice of TPT depends on the confirmed or presumed susceptibility pattern of the source patient and the possibility of other drug interactions. In case of exposure to drug-susceptible TB, 6 months of isoniazid (including in HIV-exposed infants on antiretroviral drugs) or 3 months of isoniazid–rifampicin combination should be provided; in isoniazid mono-resistant TB exposure, a 4-month rifampicin regimen should be used, while in rifampicin-resistant (RR) or multidrug-resistant TB (MDR-TB; resistance to isoniazid plus rifampicin) exposure, levofloxacin alone or in combination with another drug with known or presumed susceptibility should be used (13, 23, 24). With exposure to MDR strains with fluoroquinolone resistance (pre-extensive drug-resistant [pre-XDR] TB), or XDR-TB (pre-XDR-TB plus resistance to bedaquiline or linezolid), TPT may not be possible and expert opinion should be sought. In infants exposed to pre-XDR/XDR-TB source patients with low-level isoniazid resistance, as conferred by an *inhA* mutation only, high-dose isoniazid at 15 mg/kg for 6 months could be used as TPT (25). Delamanid as a TPT option is currently being investigated for contacts of MDR- to XDR-TB patients (26). In all exposed/infected infants, irrespective of TPT, close long-term follow-up for at least 1 year is essential for early TB disease detection.

On completion of TPT, the Bacillus Calmette–Guérin (BCG) vaccine should be administered to all infants who did not convert TST or IGRA (or if TST/IGRA is not available) and who live in countries where BCG is part of the routine vaccine policy.

### Separation of infants from mothers and other preventive measures

In low-income settings, infants are rarely separated from their mothers after birth even if the mothers are known to still be contagious. Establishing breastfeeding and bonding with the newborn infant is important. Care for the infant includes advice on infection prevention and control measures, such as wearing a mask when handling or feeding the infant until the mother is on effective therapy for at least 2 weeks and/or sputum smear-negative for acid-fact bacilli, sleeping in a separate room, allowing ventilation in the home, and adhering to mothers' own treatment and infant's TPT (6).

Temporarily separating infants from infectious mothers until they are non-infectious may be indicated in mothers with MDR-, pre-XDR-, or XDR-TB if no TPT options are available; however, this is only possible if an alternative reliable caregiver is available (22).

With the possible exception of bedaquiline, where high concentrations were reported in only one breastfeeding infant, very little drug is excreted in breast milk, and breastfeeding is not contraindicated (14, 22, 27–29). Electrocardiogram (ECG) monitoring of breastfed infants should be done if mothers are on bedaquiline.

## Clinical presentation

Congenital TB is the result of the haematogenous spread of bacilli to the endometrium: Such spread occurs mainly in primary TB (e.g., pleural effusions) or disseminated TB (e.g., miliary TB or TB meningitis), while postnatal transmission occurs mainly from mothers or other source patients with cavitary adult-type TB (6). The Cantwell revised criteria for congenital TB can assist in distinguishing congenital TB from postnatal transmitted TB: Any young infant (<3 months) with a confirmed TB lesion and one or more of the following most likely has congenital TB: (1) presents within the 1^st^ week of life, (2) a primary hepatic complex or caseating hepatic granuloma, (3) Mycobacterial bacilli identified in the placenta or endometrial TB in the mother, or (4) excluding possible postnatal transmission by excluding TB in other contacts (30).

Infants with congenital TB may be symptomatic within the 1st days of life, but mostly present between 2 and 4 weeks of age, and could be diagnosed as late as up to 5 months of age (6, 21). Symptom onset in young infants is often acute over days, rather than chronic over weeks. Initial complaints can be non-specific and vague. The clinical picture may vary from asymptomatic to severe acute or chronic illness. Infants are often misdiagnosed with acute bacterial or viral infections. Often, several courses of antibiotics are given, with poor response (31). Given the high risk of TB disease, the non-specific clinical presentation of TB in infants, and high morbidity and mortality, there should be a low threshold for rapid referral of unwell TB-exposed neonates and young infants with *any* (acute or chronic) symptoms or signs indicative of TB. The most common symptoms and signs of congenital TB in early infancy are summarized in Table 1 (6, 21, 22, 30, 32).

**Table 1:** Symptoms and signs, types of tuberculosis, and chest X-ray features in congenital TB [adapted from Schaaf et al. (6), added information from Du et al. (20), Li et al. (21), Cantwell et al. (30), Saramba and Zhao (31)].

**Symptoms and signs**	**Occurrence**
Respiratory distress including tachypnoea	Very common (i.e., >60%)
Hepatomegaly	
Fever (usually low grade)	
Cough may be acute or chronic (specifically >2 weeks of age)	Common (i.e., 40–60%)
Prematurity/low birth weight	
Failure to thrive (growth failure)	
Splenomegaly	
Poor feeding	Frequent (i.e., 25–40%)
Abdominal distension (including ascites)	
Irritability and lethargy	Infrequent (i.e., 10–25%)
Peripheral lymphadenopathy	
Sepsis syndrome	
Seizures	
Skin papular/pustular or ulcerative lesions	Rare (i.e., < 10%)
Jaundice (obstructive)	
Otorrhoea/mastoiditis	
Wheeze or stridor	
Apnoea or cyanosis attacks	
Vomiting	
Facial nerve palsy	
Shock	
Hemophagocytic lymphohistiocytosis	
**Main types of tuberculosis (can co-exist)**	
Pulmonary TB including intrathoracic lymph nodes	>90%
Miliary TB	25–35%
Abdominal TB (hepatic, splenic, peroneal lesions and ascites, intra-abdominal nodes)	20–40%
Peripheral lymph node TB	10–20%
TB meningitis	10–20%
Ear/mastoid TB	5– < 10%
Skin TB	5– < 10%
**Chest x-ray features**	
Alveolar pneumonic opacification	30–40%
Miliary infiltrates	30–40%
Bilateral bronchopneumonic infiltrates	15–20%
Intrathoracic lymph nodes	10–15%
Pleural effusion	< 5%
Cavities	< 5%
Normal chest x-ray	5–10%

## Diagnostic approach

Awareness of TB as a possible diagnosis and a high index of suspicion for TB is essential, especially in high TB (and HIV)-incidence settings. An algorithmic approach for neonates exposed to TB is proposed in Figure 1.

TB should always be considered in neonates and young infants if mothers (or other source patients) have TB, particularly in infants with pneumonia not responding to antibiotics, unremitting fever and/or hepatosplenomegaly with or without jaundice, abdominal distension with ascites, or high lymphocyte count in cerebrospinal fluid (CSF).

TB in infants is often diagnosed by a constellation of history, clinical features, and special investigations. In the history, *in vitro* fertilization and contact with an adult with TB, particularly the mother are red flags (33, 34). The history and other assessments are put together like pieces of a puzzle to decide whether a young infant has TB or not (35). However, the threshold for treating young infants for TB should be low, especially if the mother/caregiver has TB.

Clinical evaluation should assess growth (e.g., prematurity, small for gestational age, low birth weight, weight gain over time) and a full general examination and systems assessment (Table 1). Postnatal TB may differ in presentation from congenital TB: Postnatal TB often presents with cough, tachypnoea, wheeze, or stridor, while congenital TB patients more often present with acute respiratory distress, hepatomegaly, and/or abdominal signs; other presenting symptoms and signs such as poor weight gain are similar (6). Rare late presentations could even include osteoarticular TB (36).

Chest X-ray (CXR) is the most common imaging modality; CXR findings of congenital TB are summarized in Table 1. CXRs in postnatal acquired TB can look similar to congenital TB but mediastinal lymphadenopathy, large airway compression, lobar or unilateral hyperinflation, or collapse due to partial or complete large airway obstruction by enlarged mediastinal lymph nodes, respectively, and Ghon foci are more common in postnatal acquisition (6, 12). Where available, chest computed tomography (CT) scans are often used for better identification of intrathoracic lymph nodes and differentiating TB from other conditions including congenital abnormalities (20, 37).

Abdominal ultrasound is important in presumed congenital TB; ultrasound can identify hypoechoic lesions in the liver and spleen, confirm ascites in infants with abdominal distension, and identify intra-abdominal or retroperitoneal lymphadenopathy (6). Ultrasound-guided biopsy from hepatic lesions may confirm the diagnosis if microbiologically positive or granulomatous lesions are found (38). Abdominal CT scans are sometimes advised (21) but are unlikely to yield more information than abdominal ultrasound in congenital TB patients.

Central nervous system (CNS) TB, such as TB meningitis and tuberculomas, should be excluded**—**lumbar puncture for obtaining CSF for chemistry, cell count, and microbiological testing is important in all young infants presenting with presumed TB (39). Brain CT or magnetic resonance imaging (MRI) scans are important if CNS involvement is considered or if CXR shows miliary TB.

Tests of *M. tuberculosis* infection [tuberculin skin test (TST) and interferon-gamma release assay (IGRA)] can be helpful but are less often positive in young infants as it can take 6–8 weeks to convert to positive after becoming infected.

Microbiological confirmation should be sought wherever possible. Asymptomatic newborn infants may yield positive microbiological tests from gastric aspirates most likely due to ingestion of infected amniotic fluid (6, 21, 40). Specimens should be obtained from any affected site, such as respiratory secretions (gastric aspirates, tracheal aspirates, induced sputum, and bronchoalveolar lavage or stool), CSF, pleural or ascitic fluid, urine (if disseminated TB), ear swabs, and biopsy specimens of mainly lymph nodes, as well as liver and skin where feasible. Although smear-microscopy for acid-fast bacilli (AFB) may be positive, the nucleic acid amplification-based rapid molecular tests (NAATs) for *M. tuberculosis* complex (e.g., Xpert MTB/RIF Ultra and Truenat assay) are much more sensitive. Obtaining repeated specimens for testing may be necessary to confirm the diagnosis (21). Specimens should be sent for mycobacterial culture as drug susceptibility testing (DST) of children's specimens is often only confirmed on cultured isolates through molecular or phenotypic DST. The microbiological confirmation rate of *M. tuberculosis* in young infants with TB is generally high, typically 70–80% (6, 41). This is likely because of high bacillary load due to late identification of disease or uncontrolled multiplication of bacilli in the absence of a well-developed immune system. There are no data on the use of Xpert MTB/RIF Ultra on stool or urine lipoarabinomannan (LAM) in congenital TB.

To confirm congenital TB, examining the placenta may be helpful (42), and CXR and sputum (or other relevant specimen) microbiological examination for *M. tuberculosis* should be performed in the mother if not already done. In addition, an endometrial biopsy of the mother as a likely source patient is necessary if other assessments are negative (43).

## Treatment

Prompt treatment initiation is essential when TB is presumed in any neonate or young infant. Some experts advise full treatment if culture (or NAAT) for *M. tuberculosis* is positive in a young infant even if the infant is clinically well and CXR is normal (44).

Pharmacokinetic studies of first-line antituberculosis drugs in young infants are limited. Studies show that isoniazid concentrations at the current WHO-recommended dose of 7–15 mg/kg/day are adequate, but in preterm and low birth weight infants where the infant's N-acetyltransferase-2 (NAT-2) acetylation enzyme system may be immature and of slow or intermediate acetylator status, 10 mg/kg/day should not be exceeded if therapeutic drug monitoring is not available (45, 46). The current recommended rifampicin dose of 10–20 mg/kg seems too low, as very low peak concentrations (C_max_) and area under the time–concentration curves (AUCs) are reported, particularly when using liquid formulations (47, 48). Current pyrazinamide dosing at 30–40 mg/kg/day is adequate (47, 48). The ethambutol C_max_ and AUC are generally very low at the current recommended doses of 15–25 mg/kg/day; however, the risk for optic neuritis precludes higher doses (48). Ethionamide doses of 20 mg/kg are considered safe and effective across all ages of children, but there are few data in neonates (49). Pharmacokinetics of almost all second-line drugs are limited, but recent (2022) WHO-published provisional dosing recommendations for second-line drugs, including bedaquiline and delamanid, are based on available pharmacokinetic data and modeling (23).

Treatment regimens of infants with congenital or postnatal acquired TB are similar to childhood TB treatment: however, the new 4-month regimen for non-severe drug-susceptible TB is not recommended for infants of <3 months of age (23, 50). The site and severity of the disease as well as the source case or infant's *M. tuberculosis* isolate DST result should be considered when making treatment decisions. A combination of isoniazid, rifampicin, and pyrazinamide with (21) or without (37) a fourth drug, such as ethambutol, an aminoglycoside (streptomycin or amikacin), levofloxacin, ethionamide, or even linezolid (20), has mainly been used for the intensive phase in drug-susceptible TB, with a continuation phase of isoniazid and rifampicin ranging from 4 to 10 months depending on disease severity and response to treatment (6, 21, 37). The aminoglycosides should be avoided because of the risk of permanent hearing loss and the need to give it parenterally. Despite concerns about optic neuritis, ethambutol at the current recommended dose is rarely toxic, but visual acuity cannot be evaluated. Ethionamide or levofloxacin is preferred because of the high rate of miliary TB and CNS involvement**—**in such cases, the intensive phase with all four drugs should be continued throughout treatment (23, 51). Baseline liver function tests should be done, as both TB and other conditions may affect the liver in young infants, which may be misinterpreted as drug-related hepatotoxicity; liver function tests should be carefully monitored in young infants receiving hepatotoxic drugs (52).

All infants co-infected with HIV require urgent initiation of antiretroviral treatment (ART) to prevent early HIV-associated mortality (53). TPT or TB treatment should not delay initiation of ART although regimen choice may be challenging in the youngest and smallest infants. Currently, WHO recommends initiation of ART within 2 weeks, unless meningitis is present (54). All co-infected infants of more than 4 weeks of age should receive co-trimoxazole preventive therapy against *Pneumocystis jirovecii* pneumonia, irrespective of CD4 count (54). Pyridoxine levels were consistently low in children living with HIV; therefore, these children should receive pyridoxine to prevent isoniazid toxicity (55).

Infants in contact with mothers or other source patients with drug-resistant TB should be managed as being infected or having developed disease with the same resistant strain; therefore, they should be treated according to the DST result of the adult source patient and not wait for the infant's own culture and DST results (56). However, microbiological confirmation and DST on the infant's own *M. tuberculosis* strain should be attempted to confirm drug susceptibility or resistance.

Corticosteroids are indicated in infants with TB lymph node compression of the large airways or in TB meningitis.

After completion of treatment, BCG vaccination should be considered in infants living in high TB-burden settings if the TB diagnosis was unconfirmed, including infants living with HIV who are clinically and immunologically stable on ART (CD4 > 25%) (57). Clinical follow-up for recurrence of TB, either relapse or reinfection, and post-TB disease is important (58).

## Outcome

Mortality in congenital TB remains high at 15–50% (6, 21); however, some case series report 100% survival, which may be due to publication bias (presenting only patients who survived) or early diagnosis and treatment. Outcomes in published cohorts of infants of <3 months of age, which likely include mainly postnatal acquired TB but also some congenital TB patients, have found mortality between 11 and 13% (6, 43, 59).

Pulmonary TB in infants may have a long-term effect on lung health outcomes, and neurodevelopmental delay or neurological sequelae may follow severe TB or TB meningitis. Clinical evaluation and CXR (if severe lung involvement) should be done at the end of treatment to exclude post-TB disease, and follow-up and multidisciplinary management should be provided where indicated (58).

## Discussion

Neonates and young infants are at high risk of developing TB after *M. tuberculosis* infection, but the diagnosis is often missed, or TB is diagnosed late, leading to high morbidity and mortality. A high index of suspicion for TB in mothers and their infants is required, especially in high TB or TB/HIV-burden settings. TB in neonates/infants may have an atypical, often acute presentation.

Diagnosis of TB in neonates and infants is challenging. The role of tests such as IGRA, urinary LAM, and stool NAAT in this age group needs further evaluation. Furthermore, alternative imaging modalities, such as chest CT, should be evaluated in infants with pulmonary infiltrates not responding to antibiotics, who have complicated intrathoracic lymph node disease or possible congenital abnormalities.

If TB is diagnosed in an infant, the mother should primarily be screened and endometrial TB considered, especially in neonatal TB. TB bacteriological investigations including DST in the mother (or other source patient) should guide management of the infant in the absence of microbiological confirmation in the infant. If TB infection or disease is presumed in an infant, TB preventive therapy and treatment, respectively, should not be delayed, as progression to TB disease or disseminated TB is very high, with high morbidity and mortality.

Although enrolment may be complex and drug formulations limited, neonates and young infants should be included in pharmacokinetic and safety studies evaluating new and better TB drugs and regimens, including new strategies, as they become available (60–62). Although optimal dosing of antituberculosis drugs in young infants is still being evaluated, dosing recommendations exist, and drugs should be administered accordingly and adjusted with weight gain (23). Further studies of the transmission of drugs through breast milk are needed.

Finally, it is important to also screen other children and adults in the household if a mother has TB.

## Author contributions

HS wrote first draft and AB and HR reviewed the manuscript and gave critical input. All authors agreed on the final manuscript.
